# Supplementary material for: DivIVA controls the dynamics of septum splitting and cell elongation in Streptococcus pneumoniae
Source: mBio. 2024 Sep 17;15(10):e01311-24. doi: 10.1128/mbio.01311-24 (PMC11481917; doi:10.1128/mbio.01311-24)
Supplement: Supplemental material — Fig. S1 to S5 and Tables S1 and S2. [file mbio.01311-24-s0001.docx]

**Supplementary Information**

**DivIVA controls the dynamics of septum splitting and cell elongation in *Streptococcus pneumoniae***

Jennyfer Trouve, André Zapun, Laure Bellard, Dimitri Juillot, Anais Pelletier, Celine Freton, Morgane Baudoin, Rut Carballido-Lopez, Nathalie Campo, Yung-Sing Wong, Christophe Grangeasse and Cecile Morlot

**Supplementary Figures**


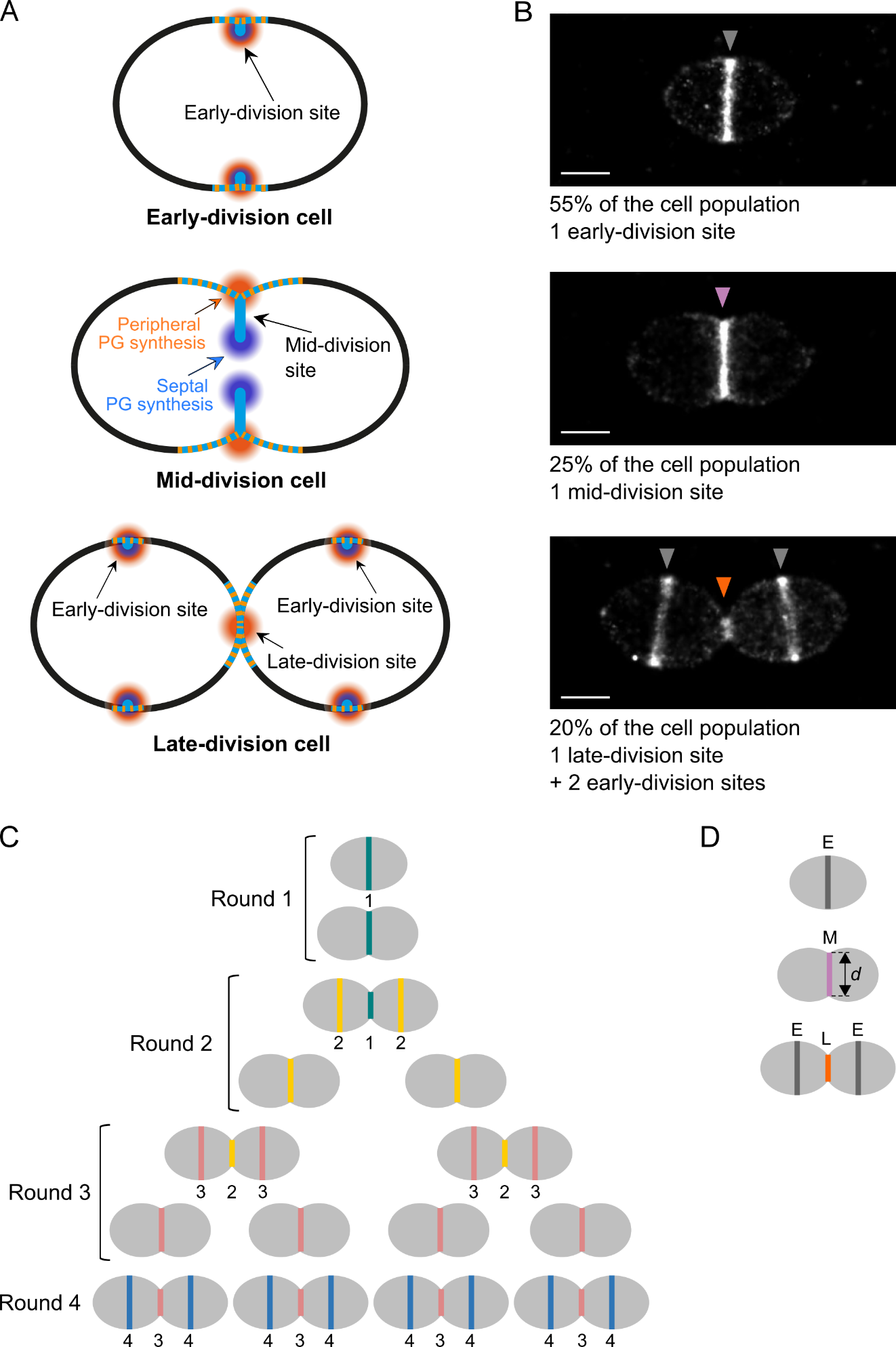


**Figure S1. Peptidoglycan synthesis in wild-type *S. pneumoniae.* A.** Model of PG growth and remodeling at three main stages of the cell cycle in ovococci. The septal PG (light blue) is synthesized by the divisome (dark blue pastille) at mid-cell, at the leading edge of the growing septum. The peripheral PG (light orange) is inserted by the elongasome (dark orange pastille) at the periphery of the septum, while this one is split and converted into lateral wall. At the beginning of the cell cycle (early-division cells), the divisome and elongasome co-localize within regions separated by less than 30 nm at early division sites. As septal PG is cleaved slower than it is synthesized, a septum forms progressively (mid-division cells), leading to the spatial separation of the septal and peripheral synthesis sites at mid-division sites. At the end of the cell cycle (late-division cells), the septum is closed but the elongasome continues synthesizing peripheral PG at the late-division site until full septum cleavage. At this stage, two new early PG labeling rings appear at the division sites of the future daughter cells. **B.** Representative dSTORM images of wild-type *S. pneumoniae* cells, corresponding to the cell cycle stages illustrated in panel A. The percentage of early, mid and late classes among the cell population (n = 231) is indicated, together with the number and category of division sites displayed by each class of cell. Scale bars, 500 nm. **C.** Schematic illustration of the successive generations observed in wild-type *S. pneumoniae* cells. First, second, third and fourth generations are respectively indicated with teal, yellow, pink and blue colors. **D.** Schematic diagram of pulse-labeled wild-type cells lying along their longitudinal axis. The dark grey, purple and orange bands correspond respectively to early- (E), mid- (M) and late-division (L) pulse-labeling patterns, defined by their respective diameter (*d*). Data and illustrations shown in panels A to D are adapted from (1).

.

**
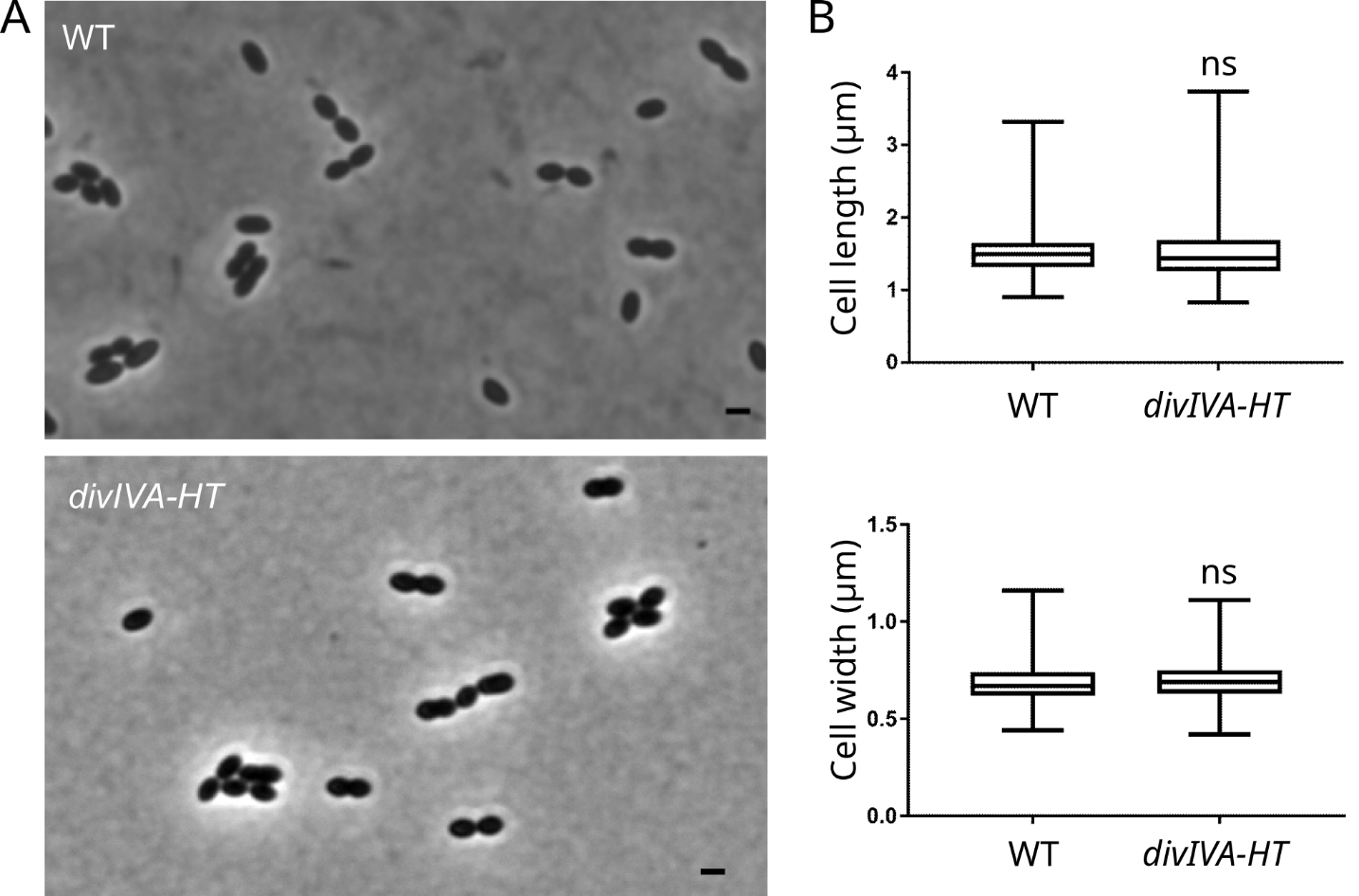
Figure S2. Characterization of the cell morphology of the *S. pneumoniae* strain expressing the DivIVA-HT fusion protein. A.** Phase contrast images of exponentially growing wild-type (WT) *S. pneumoniae* and a strain producing a DivIVA-HT fusion protein from the endogenous *divIVA* site. Scale bars, 1 µm. **B.** Distributions of cell length and width among the cell population in WT (n = 620) and *divIVA-HT* (n = 717) strains are represented with box plots showing the interquartile range (25^th^ and 75^th^ percentile), the median value and whiskers for minimum and maximum values. P-values from the unpaired U test of Mann-Whitney show no significant difference in the cell length (p-value = 0.77) or in the cell width (p-value = 0.29) between the WT and *divIVA-HT* strains.

**
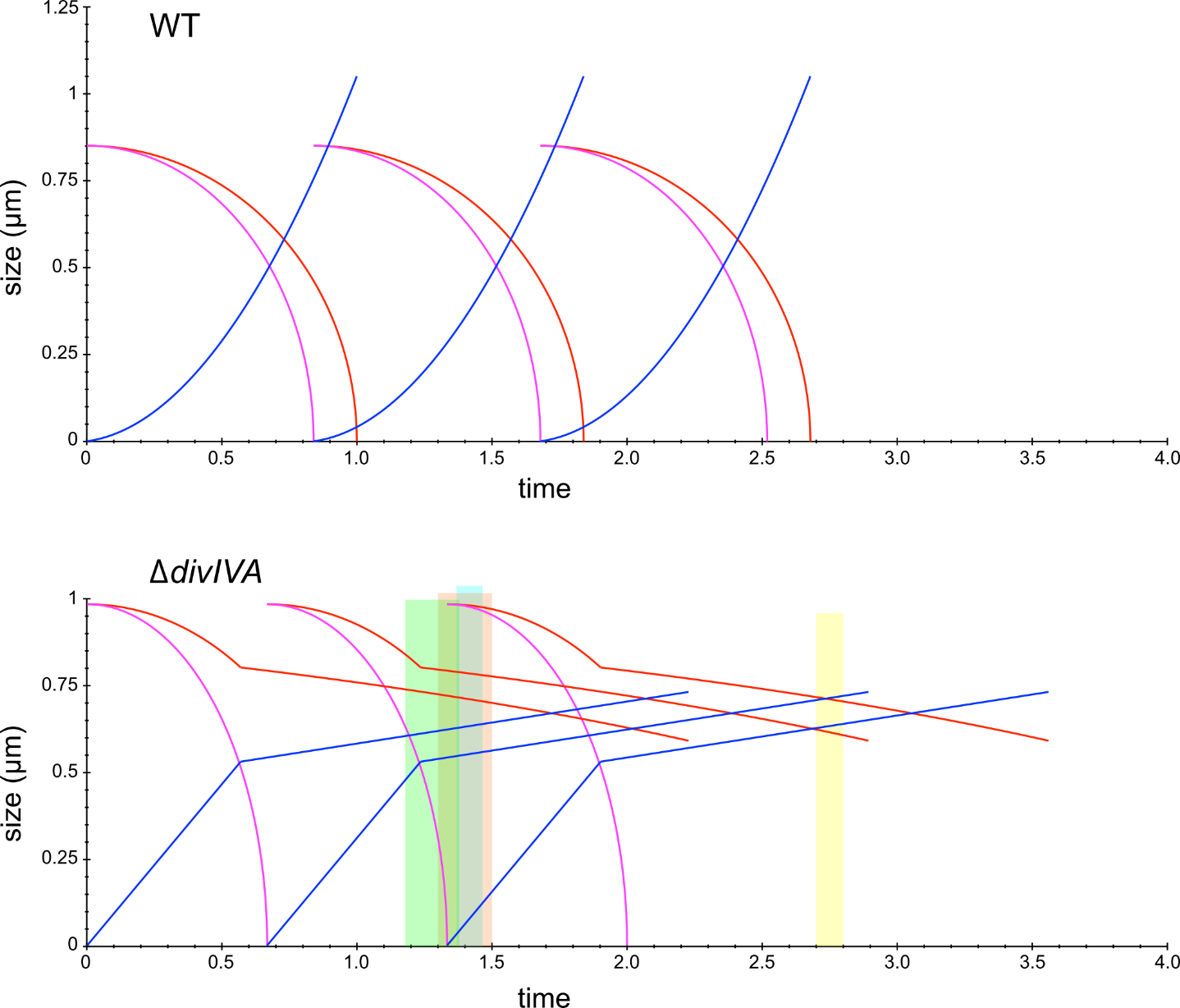
Figure S3. Modeled evolution of the cell parameters of WT and Δ*divIVA* cells.** Blue, cell length *l*; red, outer septal diameter; magenta, inner septal diameter with the chosen parameters given in Table 1. The time unit is that required to complete a division for the WT strain, or that it would take Δ*divIVA* cells to complete a division if it were not slowed and stopped prematurely. The colored rectangles represent the timing of the labeling pulses of Fig. 3A (green), 3B (orange), 3C (cyan) and 3D (yellow).


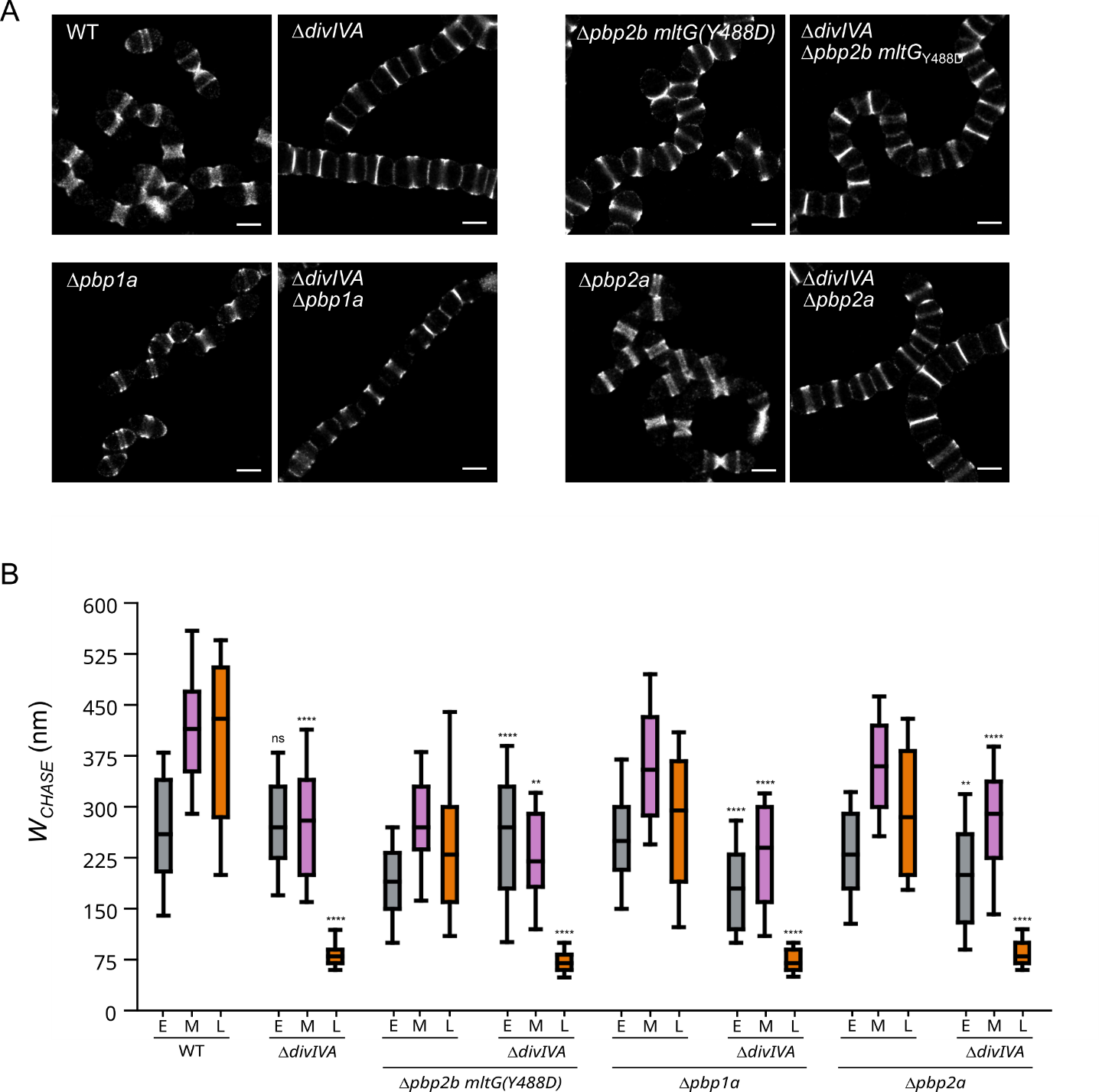


**Figure S4. Effect of *divIVA* gene deletion on pulse-chase labeling patterns obtained with otherwise wild-type and *pbp* mutant backgrounds. A.**  Fields of wild-type (WT), Δ*divIVA*, Δ*pbp2b* *mltG(Y488D)*, Δ*divIVA* Δ*pbp2b* *mltG(Y488D)*, Δ*pbp1a*, Δ*divIVA* Δ*pbp1a*, Δ*pbp2a* and Δ*divIVA* Δ*pbp2a* cells treated with the pulse-chase labeling protocol and observed by dSTORM. Scale bars, 1 µm. **B.** Distributions of *W_Chase_* of the pulse-chase labeling patterns observed in panel A (WT, n(E) = 132, n(M) = 123 and n(L) = 46; (Δ*divIVA*, n(E) = 120, n(M) = 57 and n(L) = 172 cells; Δ*pbp2b mltG*(Y488D), n(E) = 148, n(M) = 84 and n(L) = 65 cells; Δ*divIVA* Δ*pbp2b mltG(Y488D)*, n(E) = 132, n(M) = 50 and n(L) = 120 cells; Δ*pbp1a*, n(E) = 140, n(M) = 116 and n(L) = 42 cells; Δ*divIVA* Δ*pbp1a*, n(E) = 121, n(M) = 71 and n(L) = 109 cells; Δ*pbp2a*, n(E) = 109, n(M) = 108 and n(L) = 40 cells); Δ*divIVA* Δ*pbp2a*, n(E) = 112, n(M) = 42 and n(L) = 146 cells). Data are represented with box plots showing the interquartile range (10^th^ and 90^th^ percentile), the median value and whiskers for minimum and maximum values. P-values from the U test of Mann-Whitney between different data sets are indicated with double asterisks when p-values < 0.01, triple asterisks when p-value < 0.001, quadruple asterisks when p-values < 0.0001, or with "ns" when no significant difference is observed (p-value > 0.05).

**
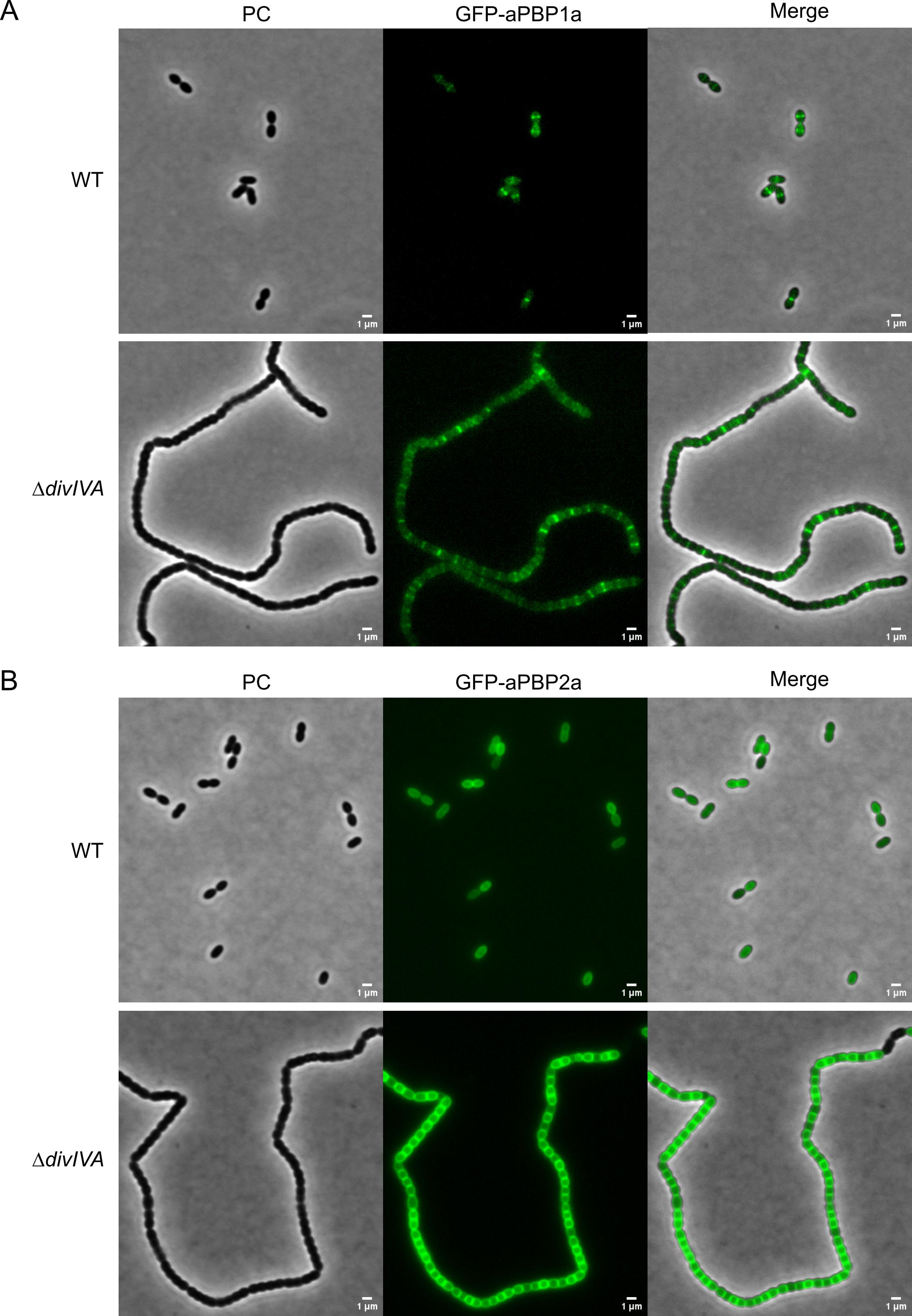
**

**Figure S5. Localization of GFP-aPBP1a and GFP-aPBP2a fusion proteins in the presence and in the absence of DivIVA. A-B** Wild-type (WT) and Δ*divIVA* *S. pneumoniae* cells expressing an ectopic copy of GFP-aPBP1a under the control of a Zn-inducible promoter (**A**), or GFP-aPBP2a under the control of a ComS-inducible promoter (**B**), were observed by conventional fluorescence microscopy. Images acquired with the phase contrast (PC) and GFP channels are shown, together with a merged image of the two channels. Scale bars, 1 µm.

**Supplementary Table 1. *S. pneumoniae* strains used in this study.**

| **Construct** | **Genotype** | | **Source** |  |
| --- | --- | --- | --- | --- |
| R800 | | *rpsL1*; *Str^R^* | (2) |  |
| sspMJ83 | | R800, Δ*divIVA* markerless; *Str^R^* | (3) |  |
| sspJT90 | | R800, Δ*pbp2b mltG(Y488D)* markerless; *Str^R^* | This study |  |
| sspCM396 | | R800, Δ*pbp1a* markerless; *Str^R^* | This study |  |
| sspCM393 | | R800, Δ*pbp2a* markerless; *Str^R^* | This study |  |
| sspJT91 | | R800, Δ*divIVA* Δ*pbp2b mltG(Y488D)* markerless; *Str^R^* | This study |  |
| sspJT92 | | R800, Δ*divIVA* Δ*pbp1a* markerless; *Str^R^* | This study |  |
| sspJT96 | | R800, Δ*divIVA* Δ*pbp2a* markerless; *Str^R^* | This study |  |
| R1501 | | Δ*comC* | (4) |  |
| R4596 | | Δ*comC*, *divIVA-HT* markerless | This study |  |

**Supplementary Table 2. Oligonucleotides used in this study.**

| **Primer name** | **Sequence** | | **Source** |  |
| --- | --- | --- | --- | --- |
| DJ8 | | caatatcaccagacttcatgcaagac | This study |  |
| DJ10 | | accaaccacggtattggtcagaac | This study |  |
| DJ41 | | cagttggcccaatgtatgaagaaccagaagt caggatctggtggagaagcagc | This study |  |
| DJ42 | | gttggacctgtcggatgcactggagttaaccagagatttccaatgtagacaacc | This study |  |
| DJ43 | | ctgctgcttctccaccagatcctgacttctggttcttcatacattgggccaact | This study |  |
| DJ44 | | gttgtctacattggaaatctctggttaactccagtgcatccgacaggtccaac | This study |  |

**Supplementary References**

1. Trouve J, Zapun A, Arthaud C, Durmort C, Di Guilmi AM, Söderström B, Pelletier A, Grangeasse C, Bourgeois D, Wong Y-S, Morlot C. 2021. Nanoscale dynamics of peptidoglycan assembly during the cell cycle of Streptococcus pneumoniae. Curr Biol CB 31:2844-2856.e6.

2. Lefevre JC, Claverys JP, Sicard AM. 1979. Donor deoxyribonucleic acid length and marker effect in pneumococcal transformation. J Bacteriol 138:80–86.

3. Fleurie A, Manuse S, Zhao C, Campo N, Cluzel C, Lavergne J-P, Freton C, Combet C, Guiral S, Soufi B, Macek B, Kuru E, VanNieuwenhze MS, Brun YV, Guilmi A-MD, Claverys J-P, Galinier A, Grangeasse C. 2014. Interplay of the Serine/Threonine-Kinase StkP and the Paralogs DivIVA and GpsB in Pneumococcal Cell Elongation and Division. PLOS Genet 10:e1004275.

4. Dagkessamanskaia A, Moscoso M, Hénard V, Guiral S, Overweg K, Reuter M, Martin B, Wells J, Claverys J-P. 2004. Interconnection of competence, stress and CiaR regulons in Streptococcus pneumoniae: competence triggers stationary phase autolysis of ciaR mutant cells. Mol Microbiol 51:1071–1086.
